# Supplementary material for: A prognostic matrix gene expression signature defines functional glioblastoma phenotypes and niches
Source: Commun Biol. 2026 Jan 5;9:18. doi: 10.1038/s42003-025-09245-8 (PMC12769572; doi:10.1038/s42003-025-09245-8)
Supplement: Supplementary file 2 — Supplementary Information [file 42003_2025_9245_MOESM2_ESM.pdf]

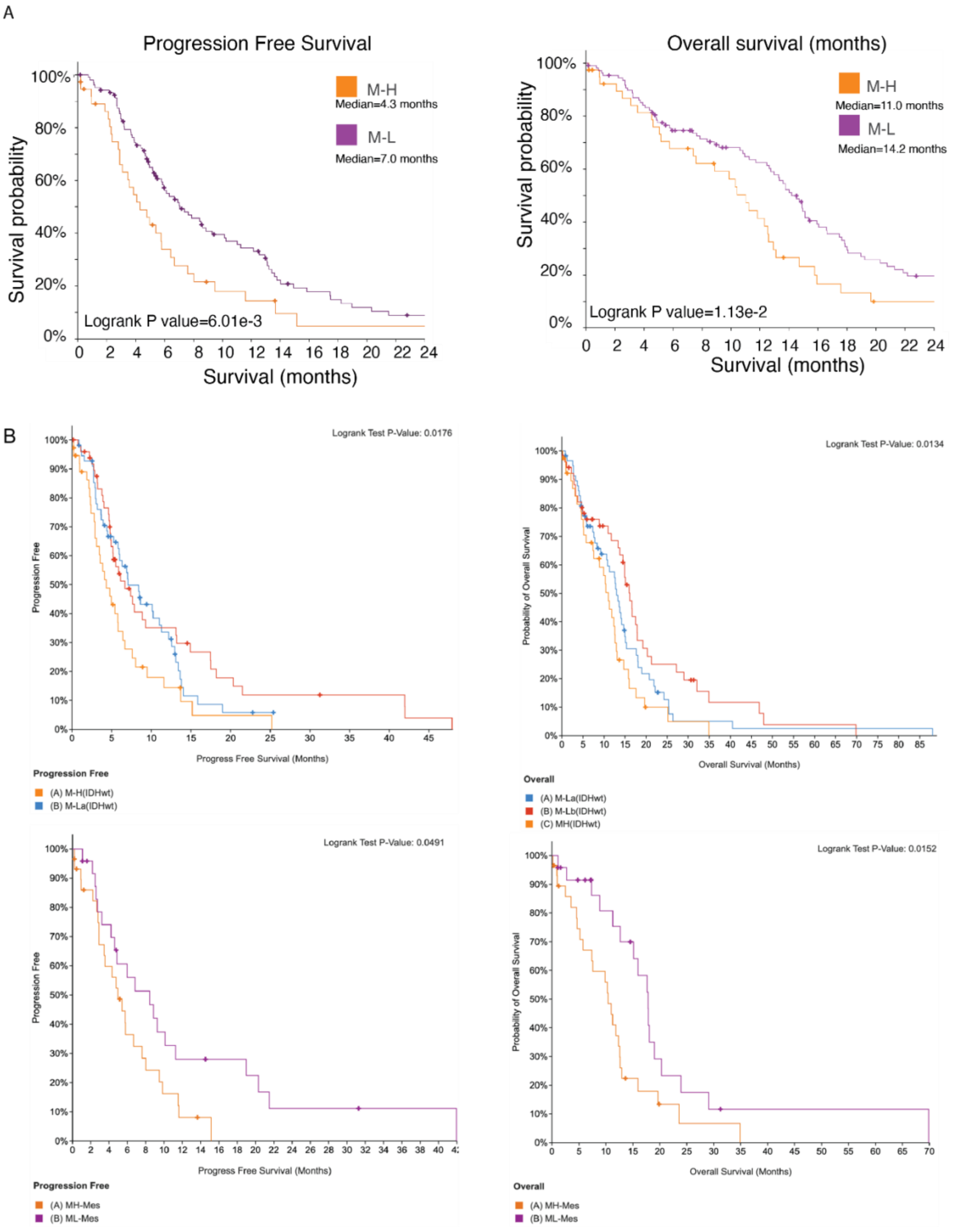
A
Progression Free Survival
Survival probability
M-H
Median=4.3 months
M-L
Median=7.0 months
Logrank P value=6.01e-3
Survival (months)
Overall survival (months)
Survival probability
M-H
Median=11.0 months
M-L
Median=14.2 months
Logrank P value=1.13e-2
Survival (months)
B
Logrank Test P-Value: 0.0176
Progression Free
Progress Free Survival (Months)
Progression Free
(A) M-H(IDHwt)
(B) M-La(IDHwt)
Logrank Test P-Value: 0.0134
Probability of Overall Survival
Overall Survival (Months)
Overall
(A) M-La(IDHwt)
(B) M-Lb(IDHwt)
(C) MH(IDHwt)
Logrank Test P-Value: 0.0491
Progression Free
Progress Free Survival (Months)
Progression Free
(A) MH-Mes
(B) ML-Mes
Logrank Test P-Value: 0.0152
Probability of Overall Survival
Overall Survival (Months)
Overall
(A) MH-Mes
(B) ML-Mes

C

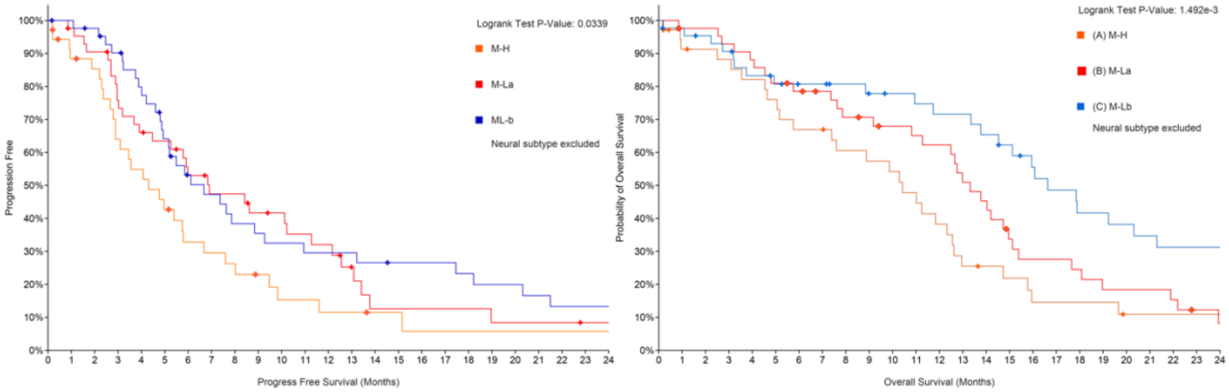

Supplementary Figure 1. The landscape of ECM composition and patient survival in glioblastoma. A. *Kaplan-Meier analysis comparing overall and progression-free survival of the M-H subgroup with the M-La and M-Lb subgroups merged as M-L.* ***B.*** *The complete and untruncated survival curves for analyses in figure 1C and D. All underlying data and statistics are identical.* ***C.*** *Kaplan-Meier analysis comparing progression free and overall survival of the M-H subgroup with the M-La and M-Lb subgroups after exclusion of samples annotated with the neural canonical subtype. Source data: TCGA GBM.*

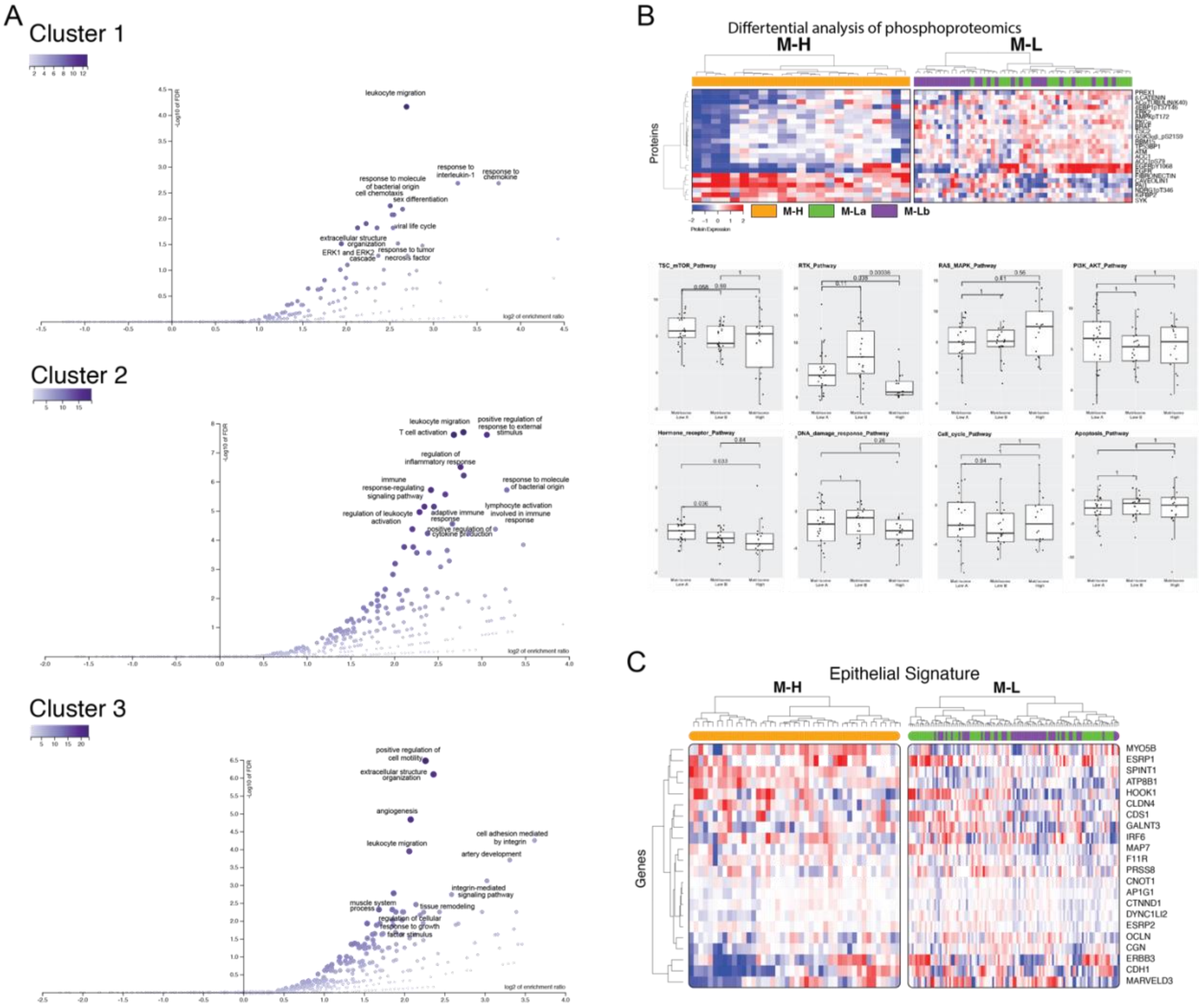

Supplementary Figure 2. The differential multi-omic landscape of matrisome-enrichment in GBM. A. *Gene set enrichment analysis of each of the clusters in Figure 2B.* ***B.*** *The heatmap of phosphoproteomic entities with significant enrichment in M-H vs. M-L groups (top). The phosphroteomics-based pathway score differences between M-H, M-La and M-Lb groups.* ***C.*** *The transcriptomic analysis of epithelial signature (see main text figure 2 for the mesenchymal signature). Source data: GBM TCGA.*

A

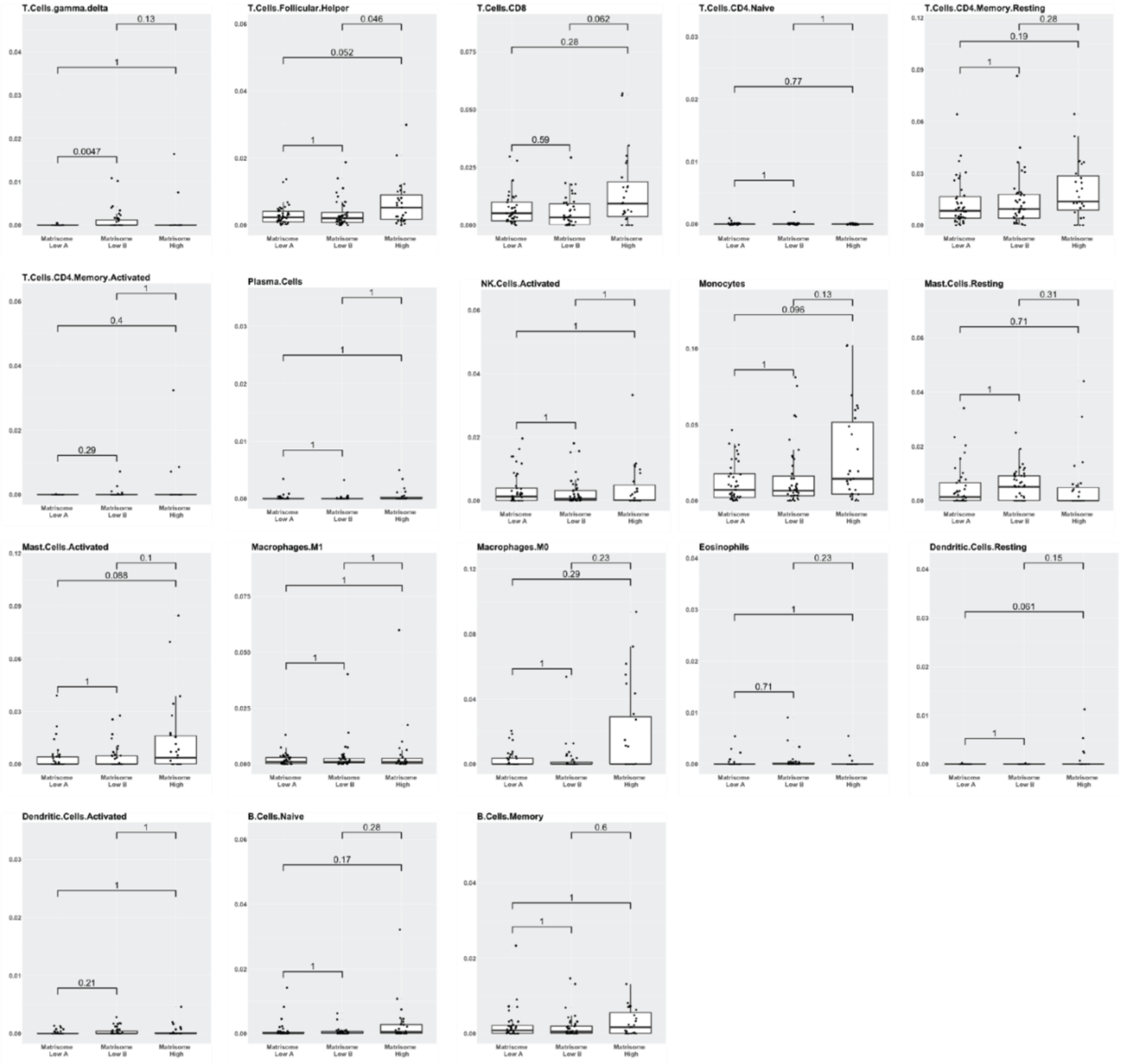
T.Cells.gamma.delta
T.Cells.Follicular.Helper
T.Cells.CD8
T.Cells.CD4.Naive
T.Cells.CD4.Memory.Resting
T.Cells.CD4.Memory.Activated
Plasma.Cells
NK.Cells.Activated
Monocytes
Mast.Cells.Resting
Mast.Cells.Activated
Macrophages.M1
Macrophages.M0
Eosinophils
Dendritic.Cells.Resting
Dendritic.Cells.Activated
B.Cells.Naive
B.Cells.Memory
Matrisome Low A
Matrisome Low B
Matrisome High

B

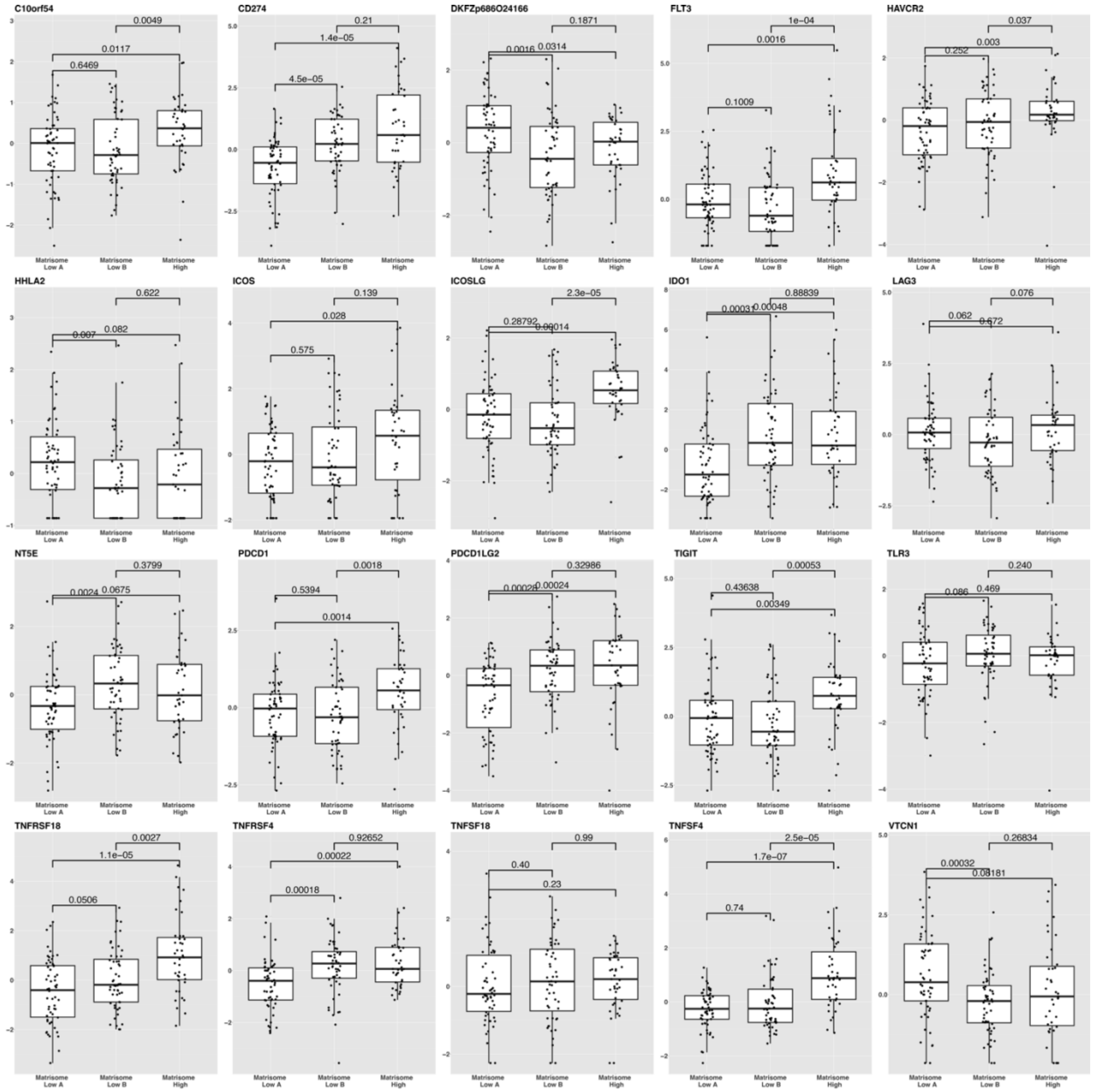

Supplementary Figure 3. The GBM tumor-immune interactions across matrisome subtypes. A. *CIBERSORT-based analysis of immune cell type enrichment in M-H vs M-La and M-Lb groups. For statistically significant enrichments of cell types and overall immune infiltration, see figure 3A.* ***B.*** *The analysis of immune checkpoint expression in M-H vs M-La and M-Lb groups. For statistically significant enrichment of receptor-ligand pairs, see figure 3B. Source data: GBM TCGA.*

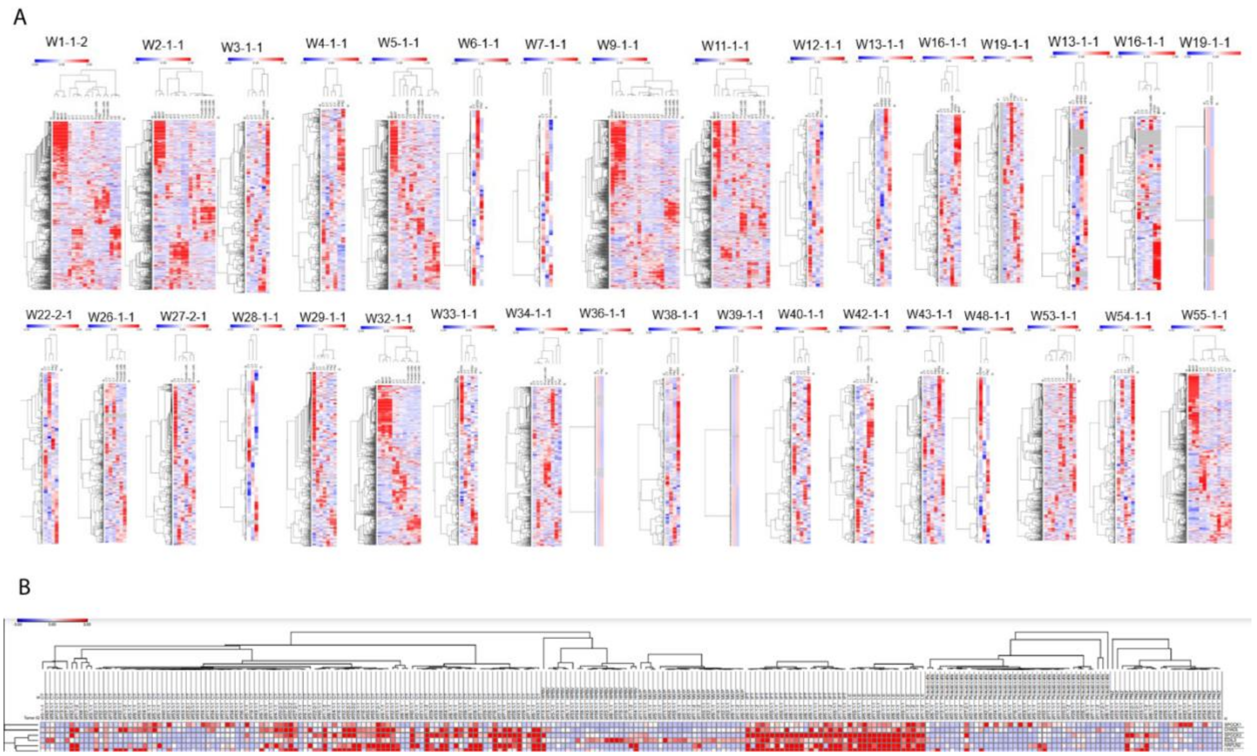

Supplementary figure 4. The spatial compartmentalization and heterogeneity of matrisome expression. A. *Hierarchical clustering analyses performed on multiple regional samples (Total=245 samples) of individual patients (n=34) show intratumoral heterogeneity based on anatomical regions. Each heatmap represents an individual patient.* ***B.*** *Supervised hierarchical clustering analysis of Oligo cell-type specific CMP genes. Source data: IvyGap.*

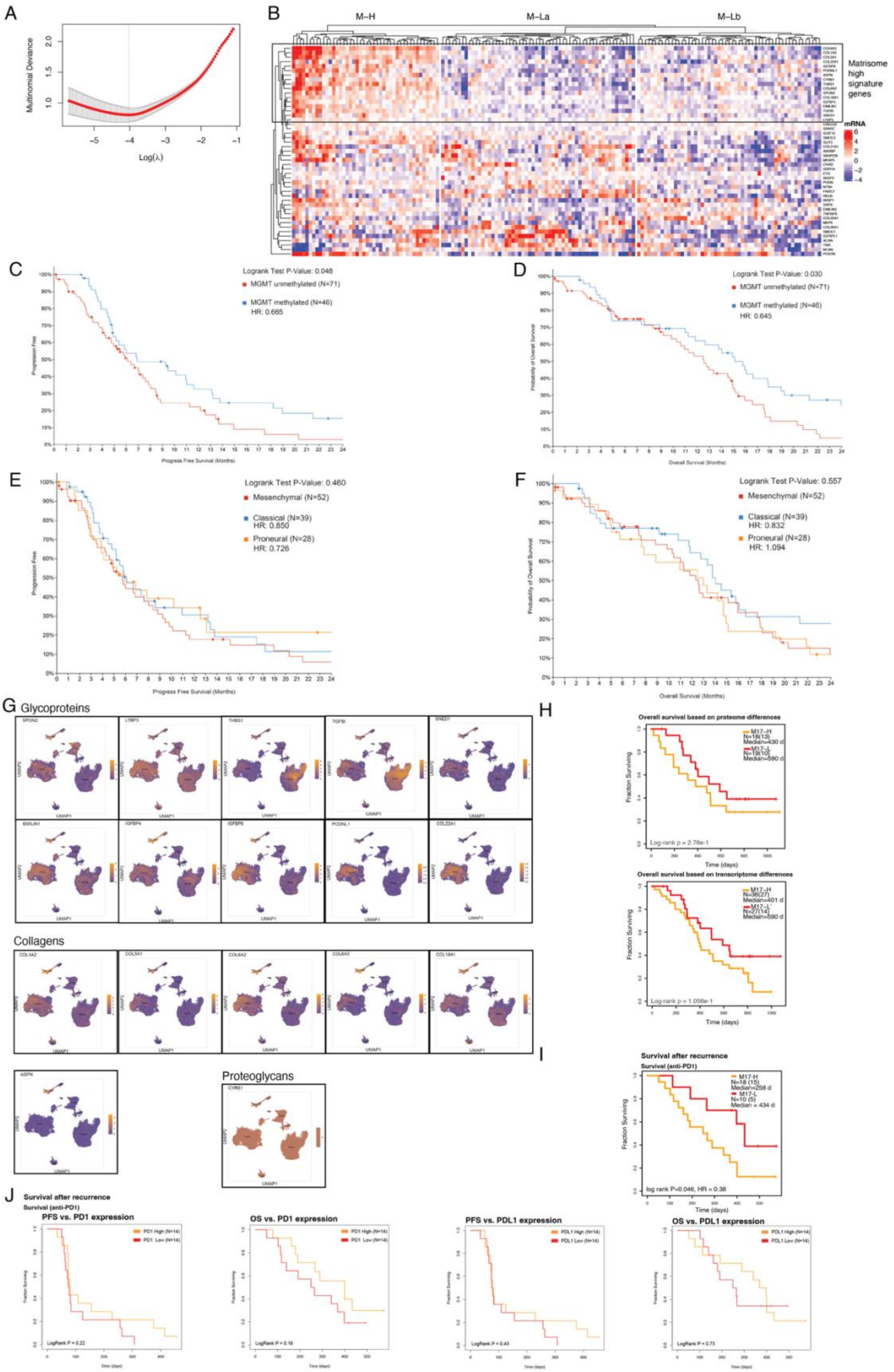
A
Multinomial Deviance
Log(λ)
B
M−H
M−La
M−Lb
Matrisome high signature genes
mRNA
C
Logrank Test P-Value: 0.048
MGMT unmethylated (N=71)
MGMT methylated (N=46)
HR: 0.665
Progression Free
Progress Free Survival (Months)
D
Logrank Test P-Value: 0.030
MGMT unmethylated (N=71)
MGMT methylated (N=46)
HR: 0.645
Probability of Overall Survival
Overall Survival (Months)
E
Logrank Test P-Value: 0.460
Mesenchymal (N=52)
Classical (N=39)
HR: 0.850
Proneural (N=28)
HR: 0.726
Progression Free
Progress Free Survival (Months)
F
Logrank Test P-Value: 0.557
Mesenchymal (N=52)
Classical (N=39)
HR: 0.832
Proneural (N=28)
HR: 1.094
Probability of Overall Survival
Overall Survival (Months)
G
Glycoproteins
Collagens
Proteoglycans
H
Overall survival based on proteome differences
Log-rank p = 2.76e-1
Overall survival based on transcriptome differences
Log-rank p = 1.056e-1
Fraction Surviving
Time (days)
I
Survival after recurrence
Survival (anti-PD1)
log rank P=0.046, HR = 0.38
Fraction Surviving
Time (days)
J
Survival after recurrence
Survival (anti-PD1)
PFS vs. PD1 expression
LogRank P = 0.22
OS vs. PD1 expression
LogRank P = 0.18
PFS vs. PDL1 expression
LogRank P = 0.43
OS vs. PDL1 expression
LogRank P = 0.73

Supplementary figure 5*. Predictive matrisome signature and response to immunotherapy.** ***A. *LASSO analysis to identify most discriminate matrisome genes. Multinomial deviance as a function of log(λ) from cross validation. The red dots and error bars represent means and standard deviations. The vertical dotted line indicates the optimal log(λ) where the multinomial deviance is minimal.* ***B.*** *Heatmap showing the selected 47 genes with 157 samples from M-H, M-La, and M-Lb subtypes. The black frame emphasizes the 17-gene matrisome signature genes that are elevated in the M-H subtype.* ***C****. Progression Free Survival of patients stratified based on MGMT promoter methylation status. Unmethylated group serves as the control for Hazard Ratio (HR) calculation. The TCGA cohort that was used in matrisome analysis is categorized based on the MGMT methylation (48 methylated samples from 46 patients, 73 methylated samples from 71 patients, 36 unclassified samples). The survival curves are truncated at 24 months while statistics is based on entire follow up period.* ***D****. Overall Survival of patients based on MGMT promoter methylation status.* ***E****. Progression Free Survival of patients stratified based on canonical (mesenchymal, proneural, classical) GBM subtypes. Mesenchymal group serves as the control for Hazard Ratio (HR) calculation. The TCGA cohort that was used in matrisome analysis is categorized based on the canonical subtypes. The survival curves are truncated at 24 months while statistics is based on entire follow up period.* ***F****. Overall survival of patients stratified based on canonical GBM subtypes.* ***G.*** *The single cell transcriptomic distribution of the genes across cell types in the matrisome signature as demonstrated through UMAP analysis.* ***H.*** *The overall survival of patients (Source: CPTAC, Wang et al, 2021) stratified as M-H' vs. M-L' based on the 17-gene matrisome protein (left) and mRNA (right) expression signature.* ***I.*** *"The recurrence to death" survival period for patients treated with anti-PD1 therapy.* ***J.*** *The progression free and overall survival times in patients with respect to immune check point (PD1 and PDL1) expression. The patients are stratified based on median PD1 (or PDL1) expression into PD(L)1 high vs PD(L)1 low groups and surviving fraction of patients in time is analyzed with a Kaplan Meier Curve. The statistical significance is evaluated with a log-rank test. Data source: (A-F) GBM TCGA; (G) scRNA transcriptomics data; (H) CPTAC; (I-J): (neo)adjuvant anti-PD1 therapy trial correlative data.*
