# Supplementary material for: A prognostic matrix gene expression signature defines functional glioblastoma phenotypes and niches
Source: Commun Biol. 2026 Jan 5;9:18. doi: 10.1038/s42003-025-09245-8 (PMC12769572; doi:10.1038/s42003-025-09245-8)
Supplement: Supplementary file 3 — Description of Additional Supplementary files [file 42003_2025_9245_MOESM3_ESM.pdf]

Description of Additional Supplementary files

File name: Supplementary Data 1

Description: A prognostic matrix code defines functional glioblastoma phenotypes and niches

Supplementary Tables

Supplementary Table 1. List of genes encoding core matrisome proteins

Supplementary Table 2. TCGA GBM cohort matrisome annotations and clinical features

Supplementary Table 3. Multivariate analysis to predict survival response based on clinical parameters

Supplementary Table 4. Clinical profiles of patients included in scRNAseq analysis

Supplementary Table 5. The proteogenomics based analysis of matrisome expression and patient survival (CPTAC cohort)

Supplementary Table 6. The analysis of anti-PD1 response and matrisome status

File name: Supplementary Data 2

Description: The source data for each figure panel is included in folders.
