# Supplementary material for: A prognostic matrix gene expression signature defines functional glioblastoma phenotypes and niches
Source: Commun Biol. 2026 Jan 5;9:18. doi: 10.1038/s42003-025-09245-8 (PMC12769572; doi:10.1038/s42003-025-09245-8)
Supplement: Supplementary file 4 — Supplementary Data 1 [file 42003_2025_9245_MOESM4_ESM.zip › Vishnoi_et-al-Korkut--SupTables-revision2/Supplementary-Tables.docx]

**A prognostic matrix code defines functional glioblastoma phenotypes and niches**

**Supplementary Tables**

**Supplementary Table 1.** List of genes encoding core matrisome proteins

**Supplementary Table 2.** TCGA GBM cohort matrisome annotations and clinical features

**Supplementary Table 3**. Multivariate analysis to predict survival response based on clinical parameters

**Supplementary Table 4**. Clinical profiles of patients included in scRNAseq analysis

**Supplementary Table 5.** The proteogenomics based analysis of matrisome expression and patient survival (CPTAC cohort)

**Supplementary Table 6.** The analysis of anti-PD1 response and matrisome status
