# Supplementary material for: A prognostic matrix gene expression signature defines functional glioblastoma phenotypes and niches
Source: Commun Biol. 2026 Jan 5;9:18. doi: 10.1038/s42003-025-09245-8 (PMC12769572; doi:10.1038/s42003-025-09245-8)
Supplement: Supplementary file 6 — Reporting Summary [file 42003_2025_9245_MOESM6_ESM.pdf]

nature portfolio

Corresponding author(s): DAPR COMMSBIO-24-8619

Last updated by author(s): Jan 9, 2025

Reporting Summary

Nature Portfolio wishes to improve the reproducibility of the work that we publish. This form provides structure for consistency and transparency in reporting. For further information on Nature Portfolio policies, see our Editorial Policies and the Editorial Policy Checklist.

Statistics

For all statistical analyses, confirm that the following items are present in the figure legend, table legend, main text, or Methods section.

n/a	Confirmed	
☐	☒	The exact sample size (n) for each experimental group/condition, given as a discrete number and unit of measurement
☐	☒	A statement on whether measurements were taken from distinct samples or whether the same sample was measured repeatedly
☐	☒	The statistical test(s) used AND whether they are one- or two-sided<br>*Only common tests should be described solely by name; describe more complex techniques in the Methods section.*
☐	☒	A description of all covariates tested
☐	☒	A description of any assumptions or corrections, such as tests of normality and adjustment for multiple comparisons
☐	☒	A full description of the statistical parameters including central tendency (e.g. means) or other basic estimates (e.g. regression coefficient) AND variation (e.g. standard deviation) or associated estimates of uncertainty (e.g. confidence intervals)
☐	☒	For null hypothesis testing, the test statistic (e.g. F, t, r) with confidence intervals, effect sizes, degrees of freedom and P value noted<br>*Give P values as exact values whenever suitable.*
☒	☐	For Bayesian analysis, information on the choice of priors and Markov chain Monte Carlo settings
☐	☒	For hierarchical and complex designs, identification of the appropriate level for tests and full reporting of outcomes
☐	☒	Estimates of effect sizes (e.g. Cohen's d, Pearson's r), indicating how they were calculated

Our web collection on statistics for biologists contains articles on many of the points above.

Software and code

Policy information about availability of computer code

Data collection	We have used TCGA, IVY-GAP GBM consortium online databases and provided all relevant information about software (cbioportal, R package, CIBERSORT, Morpheus etc.) used in manuscript text.
Data analysis	We have provided all relevant information in manuscript text. in materials & methods, and results section.

For manuscripts utilizing custom algorithms or software that are central to the research but not yet described in published literature, software must be made available to editors and reviewers. We strongly encourage code deposition in a community repository (e.g. GitHub). See the Nature Portfolio guidelines for submitting code & software for further information.

Data

Policy information about availability of data

All manuscripts must include a data availability statement. This statement should provide the following information, where applicable:

- Accession codes, unique identifiers, or web links for publicly available datasets
- A description of any restrictions on data availability
- For clinical datasets or third party data, please ensure that the statement adheres to our policy

All the data sets used in this manuscript are publicly available. The accession codes and links to repositories are provided in methods or other relevant sections.

Research involving human participants, their data, or biological material

Policy information about studies with human participants or human data. See also policy information about sex, gender (identity/presentation), and sexual orientation and race, ethnicity and racism.

Reporting on sex and gender	We have used gender as reported in online published databases used for this study.
Reporting on race, ethnicity, or other socially relevant groupings	N/A
Population characteristics	We have used gender as reported in online published databases used for this study. We have summarized all relevant information in Table 1 and supplementary information as required.
Recruitment	N/A
Ethics oversight	N/A ; De-personalized data, collected via routine methods, and shared via public databases. This study was conducted in accordance with the principles of the Declaration of Helsinki.

Note that full information on the approval of the study protocol must also be provided in the manuscript.

Field-specific reporting

Please select the one below that is the best fit for your research. If you are not sure, read the appropriate sections before making your selection.

☒ Life sciences ☐ Behavioural & social sciences ☐ Ecological, evolutionary & environmental sciences

For a reference copy of the document with all sections, see nature.com/documents/nr-reporting-summary-flat.pdf

Life sciences study design

All studies must disclose on these points even when the disclosure is negative.

Sample size	The transcriptomic analysis included RNA expression data from 157 tumor samples of 151 GBM patients used in TCGA database. IVY Gap GBM study included 110 samples across 9 patient tumors and cancer stem cell RNA-seq study included 135 samples across 31 patient tumors. For sc-RNA seq analysis, 11 GBM patients data was used as described in GSE182109
Data exclusions	For each data modality IDH1 mutations were filtered out unless otherwise stated. The RNA expression analysis of matrisome protein-encoding genes (Matrisome core genes) was initiated with 274 genes. The genes with low standard deviation across samples (s<1) were filtered out to enable classification of patients with more variant and likely discriminant transcription events.
Replication	We used multiomics approach using different database to confirm the experimental findings reported in this study.
Randomization	We have used online databases and all variables are dependent as per published databases.
Blinding	This study was conducted using de-personalized data, collected via routine methods, and shared via public databases. This study was conducted in accordance with the principles of the Declaration of Helsinki.

Reporting for specific materials, systems and methods

We require information from authors about some types of materials, experimental systems and methods used in many studies. Here, indicate whether each material, system or method listed is relevant to your study. If you are not sure if a list item applies to your research, read the appropriate section before selecting a response.

Materials & experimental systems

n/a	Involved in the study	
☒	☐	Antibodies
☒	☐	Eukaryotic cell lines
☒	☐	Palaeontology and archaeology
☒	☐	Animals and other organisms
☒	☐	Clinical data
☒	☐	Dual use research of concern
☒	☐	Plants

Methods

n/a	Involved in the study	
☒	☐	ChIP-seq
☒	☐	Flow cytometry
☒	☐	MRI-based neuroimaging

Plants

Seed stocks	N/A
Novel plant genotypes	N/A
Authentication	N/A
